# The aftermath of the pandemic: how the COVID-19 pandemic affected physical activity, fitness, health, and body fat in first-year students in Norway

**DOI:** 10.3389/fspor.2025.1719951

**Published:** 2025-12-11

**Authors:** Chiara Feldhaus, Ingeborg Barth Vedøy, Annette Løvheim Kleppang, Silje Halvorsen Sveaas, Kjersti Karoline Danielsen, Sigbjørn Litleskare, Sella Aarrestad Provan

**Affiliations:** 1 Department of Public Health and Sport Sciences, Faculty of Social and Health Sciences, University of Inland Norway, Elverum, Norway; 2 Institute of Sports and Sports Science, Karlsruhe Institute of Technology, Karlsruhe, Germany; 3 Department of Nutrition and Public Health, Faculty of Health and Sport Sciences, University in Agder, Kristiansand, Norway; 4 Centre for Health and Technology, University of South-Eastern Norway, Drammen, Norway; 5 Center for treatment of Rheumatic and Musculoskeletal Diseases (REMEDY), Diakonhjemmet Hospital, Oslo, Norway

**Keywords:** COVID-19, physical activity, lifestyle changes, first year students, adolescent health, pandemic, body composition

## Abstract

Public health measures to limit the spread of COVID-19 included restricting physical activity (PA). Here we described the impact of pandemic restrictions and reduction in PA on physical fitness and health and body composition amongst first-year students, and the associations to body fat and total PA at the end of their first year. *“*On your own feet” is a longitudinal study exploring changes in lifestyle habits amongst first-year students. Questionnaires for assessment of perceived restriction, PA behaviour and fitness and health were administered at the start and end of the first year at university. Body composition (bioelectrical impedance analysis) and total PA (Actigraph®) were recorded at both time-points. In multivariable models we identified factors associated to body fat and total PA. We included 150 students aged 18-22 years, 53% of whom reported restrictions and 34% a reduction in PA due to the COVID-19 pandemic. Students reporting restrictions had comparable fitness, health, body composition and PA level at baseline and follow-up, compared to those without restrictions. Students with reduced PA less often reported “good” fitness (30% vs. 56%, *p* < 0.001) and health (54% vs. 70%, *p* = 0.046) and had higher mean body fat percentage (27% vs. 23%, *p* = 0.009) and lower total PA (314 vs. 420 cpm, *p* < 0.001) at baseline, compared to those without reduction in PA. At follow-up, they less often reported “good” physical fitness (26% vs. 54%, *p* = 0.005), while body composition and total PA were comparable. We concluded that students who report pandemic reduction in PA may need targeted interventions to improve fitness.

## Introduction

In March 2020, the World Health Organization (WHO) officially characterised COVID-19 as a pandemic (1). The Norwegian government responded with different national measures that could be supplemented by regional or local measures imposing varying degrees of restrictions (2). These national measures in the first COVID-19 lockdown resulted in the cancellation and prohibition of fitness centres, swimming pools and organised sports activities (2). Furthermore, during this time, all teaching for university students was carried out digitally (2). The restrictions for higher education were eased in February 2021 allowing students to attend physically on university premises, and sports events could be resumed if participants came from the same municipality (3). During the ensuing months a gradual reopening occurred, and outdoor organised sport activities resumed as normal. Indoor sport activities proceeded in some areas despite prevalent infections and restrictions were gradually lifted due to high vaccination rates such as reopening of fitness centres (2, 3). Thus, COVID-19 resulted in restrictions in several aspects of adolescents life, but to a varying degree according to area of residence, level of education and individual recreational activities including physical activity (PA) (4).

An overwhelming amount of previous research concluded that PA is important in public health and associated with a lower risk for non-communicable diseases (5, 6). Globally, according to the WHO in 2016, about 81% of adolescents aged 11–17 (7) and 27.5% of 18–65-year-olds did not fulfil PA guidelines (8). From the age of 18, major benefits of PA can be gained from 150 to 300 min of moderate or 75–150 min of vigorous intensity (9). The WHO also highlights the importance for children and young adults being encouraged to participate in PA (9), stating that health-related behaviours, e.g., PA, established in early life can predict PA in later adult life (10). However, the transition from high school to university is a vulnerable phase of major importance to public health. There is evidence of a decrease in PA and changes in body weight as new lifestyle choices are established by students (11, 12). Therefore, it remains to be investigated how a new lifestyle, such as starting university and simultaneous restrictions due to the COVID-19 pandemic, may have influenced the health behaviour.

In order to examine this impact of the COVID-19 pandemic, the aims of the present study were to (1) describe the impact of self-reported pre-university pandemic restrictions and reduction in PA on organised sport activities, PA behaviour, self-reported physical fitness and health and body composition among first-year students, and (2) explore if self-reported pre-university restrictions and reduction in PA were associated with body fat and total PA at the end of their first year.

## Materials and methods

### Design and participants

**Figure 1:**
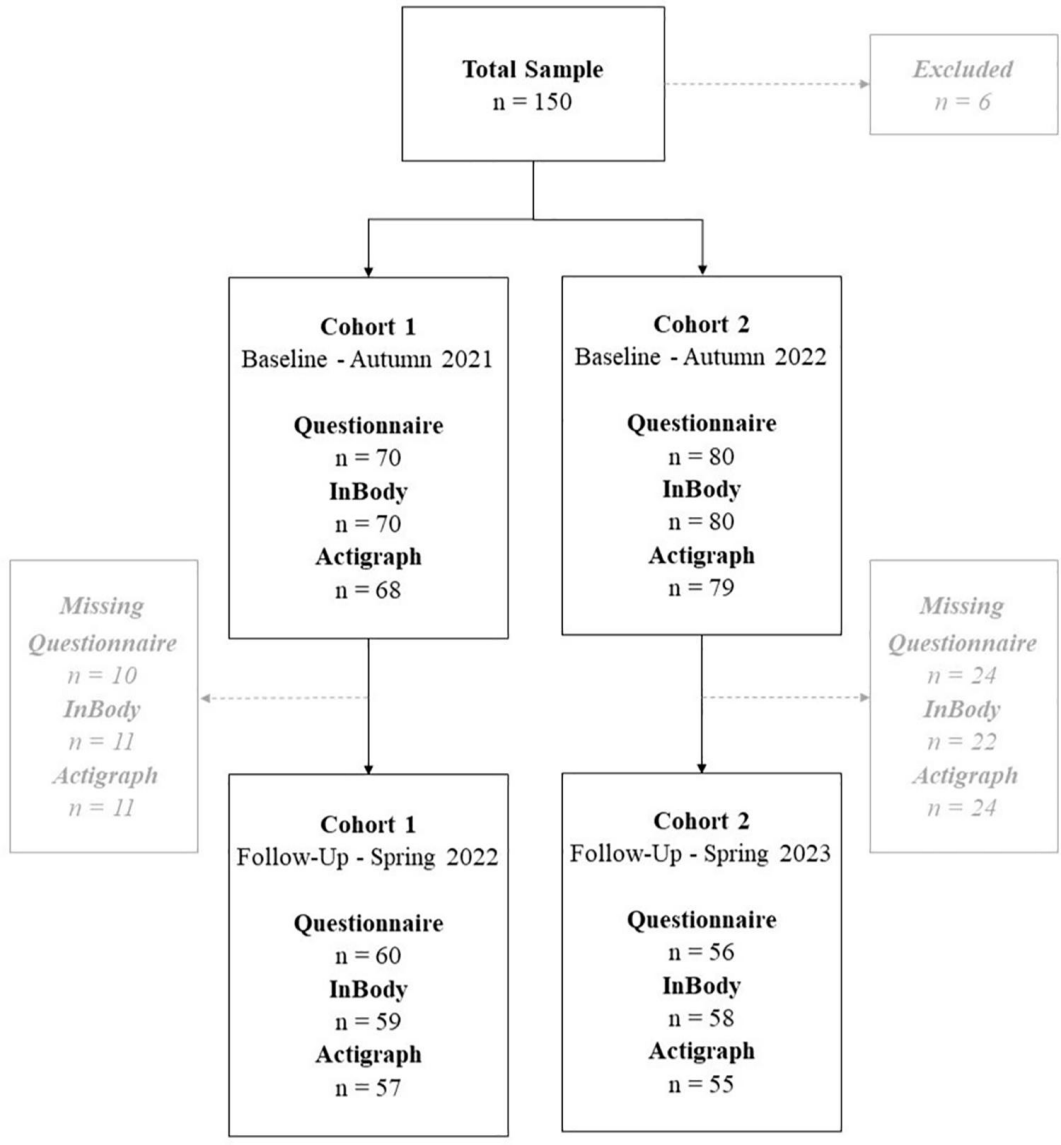
The “on your own feet” data-collection.

The study primarily investigated data from the longitudinal study “On your own feet” involving two universities in Norway (University of Inland Norway, INN, Campus Elverum; University in Agder, UiA, Campus Kristiansand) and assesses changes in lifestyle habits and physical and mental health among students during their first year at university, in two consecutive cohorts. The data collection takes place at start of the first autumn semester (baseline—start of first year) and is repeated at end of the first spring semester (follow-up—end of first year). This study consisted of two cohorts, and data were collected in August/September 2021 and April/May 2022 (cohort 1) and August/September 2022 and April/May 2023 (cohort 2) (Figure 1).

Participants were recruited through information stands and lecture visits at both universities, including first-year students aged 18–21 who no longer resided with their parents for the first time and excluded students with pacemakers or pregnancy.

The study was approved by the Regional Ethics Committee of South-Eastern Norway (255367). All participants signed informed consent forms prior to inclusion in the study.

The following variables were collected:

### Sociodemographic characteristics

Participants reported demographic information, including age (years) and place of residence at baseline and during the previous year. The former place of residence was dichotomised as urban vs. rural according to the population index from Statistics Norway from 2022 (13). The 60 largest towns in Norway were classified as urban, villages with smaller populations as rural.

### Self-reported impact of COVID-19 restrictions on PA—pre-university

At baseline, we asked students to self-report on their experience of COVID-19 restrictions in spring (March–April) before starting university studies (pre-university).

**Figure 2:**
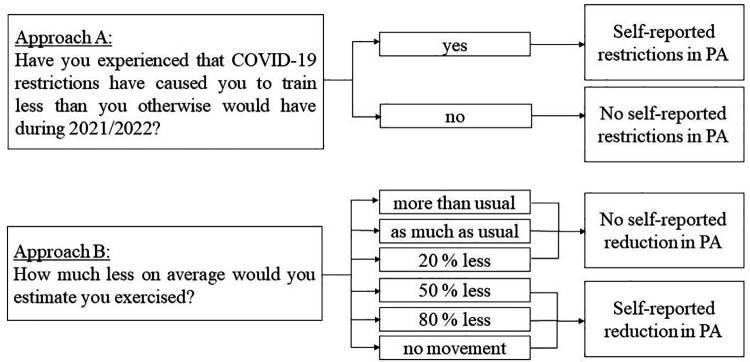
Self-reporting pre-university restrictions and reduction in physical activity during the pandemic.

The impact of COVID-19 on PA was categorised according to the response to two questions, asked retrospectively at baseline in both cohorts (Figure 2):

- Approach A: Groups were categorised according to response (“yes” or “no”) to the question “Have you experienced that COVID-19 restrictions have caused you to exercise less than you otherwise would have during 2021/2022?”, reported as “self-reported restrictions in PA” vs. “no self-reported restrictions in PA”.
- Approach B: Groups were determined by six categorical response options to the question “How much less on average would you estimate you exercised?”, ranging from “no movement” to “more than usual”, dichotomised as “self-reported reduction in PA” vs. “no self-reported reduction in PA” at the median value.

### Assessment of PA behaviour at baseline and follow-up

For objective measured PA level, participants were instructed to wear an accelerometer *(Actigraph GT3X+/GTXbt, USA)* for seven consecutive days on their waist at baseline and follow-up, except while sleeping/water activities. The device was programmed to start recording at 6.00 am the morning after receiving it and recorded data at a 10 s interval impulse (epoch). Accelerometer data were included if participants had ≥600 min of valid recordings each day. All intervals of ≥60 consecutive minutes of no recording were recorded as non-wear.

“Total PA” was used to report the overall PA level, reporting the average counts per minutes (counts*min^−1^) which is derived by dividing the total activity counts for a valid day by the sum of minutes of wear time that day for all valid days of measurement (14).

For the purpose of sensitivity analyses and evaluation of adherence to protocol, we recorded the proportion of wear days.

To assess self-reported PA behaviour at baseline and follow-up, participants were asked about their current frequency of PA by five categorical response options on a Likert scale ranging from “never” to “about every day”. Current PA intensity was assessed in three categories ranging from “without heavier breathing/sweating” to “almost fainting” and duration of PA by four categories ranging from “less than 15 min” to “more than 60 min” (15). The participation in organised sport was self-reported in six categories ranging from “never/rarely” to “daily” and we dichotomised this variable into frequent (at least 3–4 times a week, including “3–4 times a week”, “5–6 times a week”, “daily”) vs. infrequent (including “never”, “sometimes”, “once or twice a month”, “once or twice a week”).

### Self-reported fitness and health

To assess self-reported physical fitness at both time-points, participants were asked “How do you rate your own physical fitness compared to others of the same gender in your age group?”, and to assess self-reported health, we asked “How do you rate your own health compared to others of the same gender in your age group?” by five-point Likert scales. We reported responses as “bad” (“bad”, “rather bad”), “average” and “good” (“rather good”, “good”).

### Body composition

Body composition was measured by trained personnel in the lab, using direct segmental multifrequency (DSM) bioelectrical impedance (Inbody 720, Body Composition Analyzer, Biospace Co. Ltd.), after standardized procedures including no consumption of food or coffee for at least two hours before the measurement. Participants were also instructed to avoid heavy meals on that day, to use the toilet beforehand and to wear light training clothes.

Body fat (kilograms), muscle mass (kilograms) and body fat percentage were recorded. Body fat is a measure of body composition that can be distinguished from components of lean body mass (16) and was therefore chosen as the key outcome of the body composition analyses in the regression models.

### Statistical analyses

For between group differences at both time points, we compared variables across groups dichotomised according to self-reported restrictions and reduction in PA, using chi square test, student *t*-test for independent samples and Mann–Whitney *U* test, as appropriate and presented in the same way. At each item, the variable-specific number of missing is represented. Data was assumed to be missing at random and no imputations were performed.

Multivariable linear regression models were used to examine whether self-reported restrictions and reduction in PA pre-university were associated with body fat and total PA at the end of first year. In separate models, body fat (kilogram) and total PA (cpm) at end were entered as dependent variables. DAGitty [https://www.dagitty.net/] (17) was used to plot regression models in advance and choose the relevant covariates.

In adjusted models for age, gender and cohort, self-reported restrictions and reduction in PA were entered as independent variables. In separate models, body fat and total PA at baseline were also entered as covariates. Data met the required assumptions for multiple linear regression analysis. To assess potential multicollinearity, Pearson correlations among all predictor variables were inspected. No correlations, except for sex and body fat at start of year (*r* = 0.36, *p* > 0.001, Model 1.2), were found. For all analysis, statistical significance was accepted at *p* < 0.05, using IBM SPSS 25.

### Sensitivity analyses

Due to different wear days of accelerometer devices within the sample, a sensitivity analysis of the total PA regression models was carried out with an adjusted sample (4-7 wear days).

## Results

### General characteristics

A total of 150 students (69.3% female) were included, 70 participants in cohort 1 and 80 participants in cohort 2. At follow-up, 116 students attended the data-collection, 60 in cohort 1 and 56 in cohort 2, corresponding to a follow-up rate of 77.3% (Figure 1). Mean age was 19.7 (SD = 1.0) years, ranging from 18 to 22 years. At baseline, a total of 79 (53%) students self-reported restrictions in PA and 70 (47%) students reported no restrictions in PA, while 50 (34%) students self-reported a reduction in PA and 97 (66%) reported no reduction in PA (Table 1).

**Table 1:** Characteristics of samples of baseline and follow-up data. Data are presented as *n* (%) and mean (standard deviation).

Variable	Baseline *n* = 150	Follow-Up *n* = 116
Sample size of		
Cohort 1	70 (46.7)	60 (51.7)
Cohort 2	80 (53.3)	56 (48.3)
Sex		
Female	108 (69.3)	79 (68.1)
Male	46 (30.7)	37 (31.9)
Age mean (years)	19.7 (1.0)	19.7 (1.0)
Minimum	18	18
Maximum	22	22
Self-reported restrictions in PA	Valid *n* = 149	Valid *n* = 115
Yes	79 (53.0)	60 (52.2)
No	70 (47.0)	55 (47.8)
Self-reported reduction in PA	Valid *n* = 147	Valid *n* = 113
Yes	50 (34.0)	39 (34.5)
No	97 (66.0)	74 (65.5)

### Experience of restrictions according to type of PA

At baseline, both participants with restrictions in PA and those without, reported that exercising in a gym was the most frequently performed activity (35% vs. 30%), followed by exercising on their own (29% vs. 17%). Among both participants who self-reported a reduction in PA and those with no reduction in PA, reported exercising in a gym as most the frequently performed activity (16% vs. 42%), followed by exercising on their own (12% vs. 29%) (Supplementary Table S1).

### The self-reported impact of COVID-19 restrictions on PA at baseline

#### Participants who self-reported restrictions in PA pre-university

Comparing participants who self-reported restrictions in PA vs. no self-reported restriction in PA: 13% vs. 56% (*p* < 0.001) reported a reduction in PA during the pandemic. At baseline, self-reported PA level, type of affected restriction, participation in organised sports, self-reported physical fitness and health, components of body composition and total PA were comparable between participants who did vs. did not self-report restrictions in PA (Table 2).

**Table 2:** Included variables of determined groups at baseline. Data are presented as *n* (%) and mean (standard deviation).

Variable	Self-reported restrictions in PA during pandemic	No Self-reported restrictions in PA during pandemic	*p*	Missing	Self-reported reduction in PA during pandemic	No self-reported reduction in PA during pandemic	*p*	Missing
	*n* *=* *79*	*n* *=* *70*		*n*	*n* *=* *50*	*n* *=* *97*		*N*
Cohort		^a^	0.019			^a^	0.678	
Cohort 1	30 (38.0)	40 (57.1)			25 (50.0)	45 (46.4)		
Cohort 2	49 (62.0)	30 (42.9)			25 (50.0)	52 (53.6)		
Self-reported restrictions in PA during pandemic		^a^	<.001			^a^	<.001	1
Yes	79 (100.0)	0 (0.0)			10 (20.4)	66 (68.0)		
No	0 (0.0)	70 (100.0)			39 (79.6)	31 (32.0)		
Self-reported reduction in PA during pandemic		^a^	<.001	3		^a^	<.001	
Yes	10 (13.2)	39 (55.7)			50 (100.0)	0 (0.0)		
No	66 (86.8)	31 (44.3)			0 (0.0)	97 (100.0)		
Sex		^a^	0.228			^a^	0.537	
Female	58 (73.4)	45 (64.3)			36 (72.0)	65 (67.0)		
Male	21 (26.6)	25 (35.7)			14 (28.0)	32 (33.0)		
Age [years]		^b^	0.200			^b^	0.949	
	19.8 (1.0)	19.6 (1.0)			19.7 (1.0)	19.7 (1.0)		
Place of residence		^a^	0.849	5		^a^	0.996	5
Urban	39 (50.6)	35 (52.2)			25 (52.1)	49 (52.1)		
Rural	38 (49.4)	32 (47.8)			23 (47.9)	45 (47.9)		
Self-reported PA level		^c^	0.446			^c^	<.001	
Never	1 (1.3)	0 (0.0)			2 (4.0)	0 (0.0)		
Less than once a week	8 (10.1)	8 (11.4)			12 (24.0)	4 (4.1)		
Once a week	10 (12.7)	11 (15.7)			9 (18.0)	12 (12.4)		
2–3 times a week	26 (32.9)	26 (37.1)			15 (30.0)	36 (37.1)		
About every day	34 (43.0)	25 (35.7)			12 (24.0)	45 (46.4)		
Type of affected restriction		^c^	0.05	26		^c^	0.442	24
Strict training centre	25 (47.2)	41 (58.6)			26 (59.1)	41 (51.9)		
Cancelled team training	17 (32.1)	27 (38.6)			14 (31.8)	29 (36.7)		
Cancelled individual training	11 (20.8)	2 (2.9)			4 (9.1)	9 (11.4)		
Participation in organised sports		^a^	0.787	1		^a^	0.003	1
Frequent	21 (26.6)	17 (24.6)			5 (10.2)	32 (33.0)		
Infrequent	58 (73.4)	52 (75.4)			44 (89.8)	65 (67.0)		
Self-reported physical fitness		^c^	0.610			^c^	<.001	
Bad	13 (16.5)	12 (17.1)			15 (30.0)	10 (10.3)		
Average	26 (32.9)	26 (37.1)			20 (40.0)	33 (34.0)		
Good	40 (50.6)	32 (45.7)			15 (30.0)	54 (55.7)		
Self-reported health		^c^	0.218			^c^	0.046	
Bad	9 (11.4)	5 (7.1)			7 (14.0)	7 (7.2)		
Average	22 (27.8)	16 (22.9)			16 (32.0)	22 (22.7)		
Good	48 (60.7)	49 (70.0)			27 (54.0)	68 (70.1)		
Body Composition								
Body fat [kg]	15.9 (6.8)	16.9 (8.1)^b^	0.419		18.6 (8.5)	15.3 (6.7)^b^	0.010	
Muscle mass [kg]	28.7 (7.3)	29.6 (6.8)^b^	0.456		27.9 (6.5)	29.7 (7.5)^b^	0.163	
Body fat [%]	23.7 (9.1)	24.1 (9.8)^b^	0.786		26.8 (9.7)	22.5 (9.0)^b^	0.009	
PA			0.279	3			0.003	3
Total PA (cpm)	408.8 (190.8)	377.0 (158.2)^b^			340.9 (132.2)	422.1 (192.2)^b^		
PA (4–7 wear days)	*n* *=* *65*	*n* *=* *62*	0.191		*n* *=* *42*	*n* *=* *84*	<.001	
Total PA (cpm)	404.4 (174.4)	365.8 (152.6)^b^			313.6 (98.5)	419.9 (181.8)^b^		

cpm, counts per minute.

a Statistical test performed: Chi Square test.

b Statistical test performed: *t*-test for independent samples.

c Statistical test performed: Mann-Whitney *U* test.

#### Participants who self-reported reduction in PA pre-university

Significant differences were found when comparing participants who self-reported a reduction in PA vs. no self-reported reduction: 24% vs. 46% (*p* < 0.001) self-reported being active approximately every day, 30% vs. 56% (*p* < 0.001) reported good physical fitness and 54% vs. 70% (*p* = 0.046) reported good health.

Participants who self-reported a reduction in PA during the pandemic had a significant higher mean body fat percentage compared to those with no reduction: 27% vs. 23% (*p* = 0.009) and a significant lower mean total PA with 4-7 wear days: 314 cpm vs. 420 cpm (*p* < 0.001) (Table 2).

### The impact of self-reported COVID-19 restrictions on PA at follow-up

#### Participants who self-reported restrictions in PA pre-university

At end of first year, self-reported PA level, intensity and duration of activity, self-reported physical fitness and health, body composition and total PA were comparable between participants who did vs. did not self-report restrictions in PA (Supplementary Table S2).

#### Participants who self-reported reduction in PA pre-university

Comparing participants who self-reported a reduction in PA vs. no self-reported reduction in PA: 69% vs. 78% (*p* = 0.041) described the intensity of activity as “out of breath or sweating” and 26% vs. 54% (*p* = 0.005) reported “good” physical fitness at follow-up, differing significantly. At end of first year, self-reported PA level, self-reported health, components of body composition and total PA were comparable between participants who reported reduced PA pre-university (Supplementary Table S2).

### The association between self-reported restrictions and reduction in PA on long-term body fat and total PA

In multivariable models, no significant associations were found between self-reported restrictions or reduction in PA at baseline and body fat at the end of first year with consideration of sex, age and cohort. Additionally, adjusting for body fat at baseline did not change the main result. Furthermore, no significant association was found between self-reported restrictions or reduction in PA at start and PA level at end of the first year after adjustments sex, age and cohort. In either exposure, no associations were found when additionally adjusted for PA level at baseline (Table 3). The sensitivity analyses conducted on the total PA models revealed no deviations.

**Table 3:** Multivariable linear regression models of associations between pre-study restrictions/reduction to PA and end of first year body fat and total PA.

Approach	Body fat Model 1.1	Body fat Model 1.2	Total PA Model 2.1	Total PA Model 2.1 (adjusted sample)	Total PA Model 2.2	Total PA Model 2.2 (adjusted sample)
Self-reported restrictions in PA	−0.10 (−4.29; 1.24)	0.03 (−0.79; 1.77)	0.11 (−33.08; 115.53)	0.15 (−20.21; 127.68)	0.03 (−58.43; 78.43)	0.02 (−54.94; 69.41)
			*n* = 111	*n* = 96	*n* = 110	*n* = 95
Self-reported reduction in PA	0.09 (−1.35; 4.3)	−0.004 (−1.38; 1.23)	−0.08 (−111.5; 43.83)	−0.07 (−104.11; 50.43)	0.03 (−60.48; 85.56)	0.07 (−37.08; 92.61)
			*n* = 109	*n* = 94	*n* = 108	*n* = 93

Data are presented as standardised β-coefficient and 95% confidence intervals. Reference category is no self-reported restrictions/reductions to PA.

body fat [kg], total PA [cpm], cpm—counts per minute.

ns, not significant.

adjusted sample –sample with 4–7 wears days (sensitivity analysis).

Body fat model 1.1 adjusted by sex, cohort, age.

Body fat model 1.2 adjusted by sex, cohort, age, body fat at baseline.

Total PA model 2.1 adjusted by sex, cohort, age.

Total PA model 2.2 adjusted by sex, cohort, age, total PA at baseline.

## Discussion

The major findings of this study were that restrictions in PA during the COVID-19 pandemic did not impact on physical fitness and health, body composition and PA level at the start or at the end of the first year of students. We reported an association between self-reported pre-university reduced PA during the pandemic and reduced self-reported fitness and health at end of the first study year. Interestingly, students who self-reported a reduction in PA during the pandemic had significantly higher body fat and lower total PA at start, but not at the end of the first year.

We also found a difference with regards to PA at the start of university life between those who self-reported restrictions and self-reported reduction in PA during the pandemic pre-university. At the start of the first year, students who self-reported restrictions had comparable levels of self-reported PA to those who self-reported no-restrictions, while students who reported a reduction in PA report lower levels of PA compared to those who reported no reduction. There was no difference in students' place of residence in terms of perceptions of restrictions and reduction in PA, despite varying restrictions were implemented in urban and rural areas of Norway during the pandemic (2).

### Adjusting to a new daily life

Our findings indicated that students who typically engaged in organised sports, individual training and/or workouts in a gym, may have replaced their physical activities with alternative options as a result of the closures of organised sport and sport facilities during the lockdown (2, 3), even though they experienced restrictions. At start of the first year, those who felt restricted in PA pre-university showed PA levels (total PA of 404 cpm) which were comparable to the age group of 20- to 34-year-olds in the Kan3-study (18). This aspect is particularly important in view of the fact that health behaviour established in early life influences PA in later adult life (10), complemented by the general importance of health benefits in children and adolescents participating in PA (9).

The lack of agreement between the responses to questions regarding experienced restrictions in PA vs. a reduction in PA may be due to the difficulty of self-reporting the fluctuating impact of the pandemic. However, we may also have captured the difference between a perceived restriction, preventing a chosen activity but not prohibiting another, and a reduction in PA which may be due to a multiple of factors such as reduced mental wellbeing, low resilience to stress, in addition to the pandemic restrictions. Also, the shift to higher education represents a vulnerable phase for student's health-related behaviour (12) and may have led to a general high level of uncertainty among students (4).

By the end of the first year, students who self-reported restrictions and reduction in PA pre-university, seemed to have adapted their PA behaviour in this exceptional situation, as the total PA measured at end of first year were comparable across categories. Body composition, i.e., body fat and muscle mass were also comparable between groups who self-reported COVID-19 restrictions or reduction in PA pre-university vs. not, by the end of first study year.

These findings were in line with López-Valenciano et al. (19) who analysed the impact of COVID-19 on PA in university students. They found that students who fulfilled the recommended activity targets for their age before the restrictions, still were sufficient physically active during the lockdown period (19). The ability to adjust to life under lockdown may be understood in the context of resilience. Brewer and colleagues (20) summarised resilience as a “dynamic, contextual process focused on adaptation (to stress or change)” and also emphasised the importance due to associations between resilience and health and well-being of students (20). This indicated even though students experienced restrictions in PA, their adaption to changed conditions to be physical active is important for their health and well-being in the long run.

### PA behaviour

Using “total PA” as a dimension for PA, we found estimates of PA to be numerically below the average of the Norwegian population (18, 21), in students who reported a reduction in PA due to COVID-19 restrictions at start of the first study year, but not in students who reported restrictions to PA. Hansen et al. (21) monitored the Norwegian population across the lifespan and estimated an average activity level in 20- to 64-year-olds with 360 cpm in women and 377 cpm in men, similar values to those reported in the national survey for PA 2020-22 (Kan 3) in Norway specifically for 20- to 34-year-olds (18). By the end of the first year, our findings showed a comparable activity level in all students. Also, López-Valenciano et al. (19) reported a significant decrease in PA levels of university students during lockdown (19). Furthermore, neither pre-university restrictions nor the reduction in PA showed any significant impact on self-reported PA in the following year.

### Self-reported physical fitness and health

We could not find evidence that feeling restricted by COVID-19 measures with regards to PA made a difference in students perceived physical fitness and health, but we found an association between pre-university reduced PA and lower self-reported physical fitness and health both at the start and for self-reported physical fitness also at end of their first study year. Previous research on self-reported physical fitness, PA and other factors in young adults indicated that lower physical fitness and insufficient PA can cause high level of stress (22) and bad sleep quality (23) and emphasising the importance of a resilient behaviour in students in regard to health and wellbeing (20). We could not exclude that the relationship between reduced PA during the pandemic and later lower levels of self-reported fitness and health was mediated by individual risk factors making the individual vulnerable to negative consequences of the pandemic.

### Strengths and limitations

Strengths of the present study included using two measures for the impact of COVID-19 on PA and the collection of objectively measured PA and body composition with validated instruments and standardised data-collection procedures. We considered potential bias between self-reported and objectively measured health and fitness, especially in individuals with higher fitness levels (24). Objective measures of physical fitness were generally not conducted in the presented project “On your own feet”. COVID-19 influenced various facets of our life (4). To address the impact, we performed all analyses in young adults, i.e., first year students. Because participants were recruited through information stands and lecture visits, a selection bias cannot be ruled out. Students who voluntarily chose to participate may differ from those who did not (e.g., health behavior). In addition, the restrictions and reduction in PA were self-reported retrospectively which introduced potential bias, as participants may have not provided accurate experiences for the specific two-month time frame. In addition, these retrospective items were limited to restrictions and reduction in PA in relation to exercise training and thus did not capture the wider definition of PA and the binary yes/no item regarding PA restrictions limited further elaborations. In regard to the objective PA assessment, accelerometers were removed during sleep for reasons of discomfort for those wearing the device, which may introduced measurement bias in the wear time of the devices. Also, both cohorts were asked the same questions a year apart, while entering distinct phases of the pandemic, i.e., the extent of restrictions differed in view of further developments of the pandemic (e.g., higher vaccination coverage, less restrictive measures). This could mean potential bias in the findings of this study. We found inconsistencies between reported restrictions and reduction in PA, also highlighting potential retrospective data bias. One may speculate that differences could be partially explained by the mental state or resilience of students, although this is beyond the scope of this paper. Additional questions targeting the length and nature of lockdown could have been helpful, also considering the small sample size.

## Conclusion

We found that pre-university restrictions in PA during the COVID-19 pandemic did not impact on physical fitness, health, body composition and PA level of students at the start or at the end of the first year. However, students who reported reduced PA during the pandemic had lower physical fitness, poorer self-reported health, higher body fat percentage and lower PA levels at start of the first year, compared to those who did not report reduced PA. Further, physical fitness remained lower than comparators at the end of the first year. The study suggests that students who reported reduced PA during the pandemic may have had different experiences than those who reported restrictions and may have needed targeted interventions to improve physical fitness and resilience.

## Data Availability

The raw data supporting the conclusions of this article will be made available by the authors, without undue reservation.
