# Supplementary material for: The aftermath of the pandemic: how the COVID-19 pandemic affected physical activity, fitness, health, and body fat in first-year students in Norway
Source: Front Sports Act Living. 2025 Dec 11;7:1719951. doi: 10.3389/fspor.2025.1719951 (PMC12739646; doi:10.3389/fspor.2025.1719951)
Supplement: Supplementary file 1 [file Table1.docx]

Supplementary table 1: Proportion of participants in each group which trained frequently (at least 3 to 4 times a week) in physical activities in different contexts at start of first year.

| **Specific activities** | **Self-reported** **restrictions in PA**  n = 79 | **No self-reported** **restrictions in PA**  n = 70 | **Self-reported reduction in PA**  n = 50 | **No self-reported reduction in PA**  n = 97 |
| --- | --- | --- | --- | --- |
| Training/competing in a sports team n (%) | 12 (15.2) | 11 (15.7) | 3 (6.0) | 19 (19.6) |
| Training in a fitness centre n (%) | 27 (34.6) | 21 (30.0) | 8 (16.0) | 40 (41.6) |
| Other organised training n (%) | 3 (3.8) | 1 (1.4) | 0 (0) | 4 (4.1) |
| Exercising on your own n (%) | 23 (29.1) | 12 (17.2) | 6 (12.0) | 28 (28.9) |
| E-sports n (%) | 0 (0) | 2 (2.9) | 2 (4.2) | 0 (0) |

Other organised training includes dance, martial arts or similar

Exercising on your own includes running, swimming, cycling, hiking

E-sports includes training or competitions

Supplementary table 2: Included variables of determined groups at the end of first year (follow-up). Data are presented as n (%) and mean (standard deviation).

| Variable | Self-reported restrictions  in PA | No Self-reported restrictions  in PA | p | Missing | Self-reported reduction in PA | No self-reported  reduction in PA | p | Missing |
| --- | --- | --- | --- | --- | --- | --- | --- | --- |
|  | *n = 79* | *n = 70* |  | *n* | *n = 50* | *n = 97* |  | *n* |
| Self-reported PA level  never  less than once a week  once a week  2-3 times a week  about every day | 1 (1.7) 4 (6.7) 13 (21.7) 18 (30.0)  24 (40.0) | ^a^  0 (0.0)  3 (5.5)  6 (10.9)  21 (38.2)  25 (45.5) | 0.244 | 34 | 2 (5.1)  9 (23.1)  18 (46.2)  10 (25.6) | ^a^  1 (1.4)  5 (6.8)  11 (14.9)  20 (27.0)  37 (50.0) | 0.061 | 34 |
| Self-reported intensity of activity  without heavier breathing/sweating out of breath or sweating almost fainting | 9 (16.4) 42 (76.4) 4 (7.3) | ^a^  10 (19.6)  38 (74.5)  3 (5.9) | 0.622 | 43 | 10 (27.8)  25 (69.4)  1 (2.8) | ^a^  9 (13.2)  53 (77.9)  6 (8.8) | 0.041 | 43 |
| self-reported duration of activity  less than 15 minutes  15-29 minutes  30-60 minutes  > 60 minutes | 1 (1.8)  2 (3.5) 28 (49.1)  26 (45.6) | ^a^  0 (0.0)  5 (9.4)  25 (47.2)  23 (43.4) | 0.671 | 39 | 1 (2.6)  6 (15.8)  16 (42.1)  15 (39.5) | ^a^  1 (1.4)  1 (1.4)  35 (50.0)  33 (47.1) | 0.140 | 39 |
| Self-reported physical fitness  bad  average  good | 5 (8.4)  28 (46.7)  27 (45.0) | ^a^  3 (5.5)  26 (47.3)  26 (47.3) | 0.705 | 34 | 4 (10.3)  25 (64.1)  10 (25.6) | ^a^  4 (5.4)  30 (40.5)  40 (54.1) | 0.005 | 34 |
| Self-reported health  bad  average  good | 5 (8.3)  21 (35.0)  34 (56.7) | ^a^  4 (7.3)  20 (36.4)  31 (56.4) | 0.985 | 34 | 4 (10.3)  18 (46.2)  17 (43.6) | ^a^  5 (6.8)  23 (31.1)  46 (62.2) | 0.066 | 34 |
| Body composition  Body fat [kg]  Muscle mass [kg]  Body fat [%] | 17.4 (6.8)  29.4 (7.7)  25.0 (8.4) | ^b^  18.2 (8.8)  ^b^  30.1 (7.1)  ^b^  25.2 (10.1) | 0.554  0.609  0.933 | 31  31  31 | 18.9 (8.9)  28.4 (6.4)  26.8 (9.7) | ^b^  17.2 (7.3)  ^b^  30.4 (8.1)  ^b^  24.3 (9.0) | 0.260  0.179  0.188 | 33  33  33 |
| PA  Total PA (cpm) | 404.16 (226.7) | ^b^  377.2 (155.7) | 0.466 | 38 | 368.0 (140.3) | ^b^  402.5 (218.4) | 0.322 | 38 |
| PA (4-7 wear days)  Total PA (cpm) | *n = 50*  388.1 (211.5) | *n = 46*  ^b^  357.5 (137.4) | 0.395 |  | *n = 30*  357.5 (147.5) | *n = 64*  ^b^  380.8 (195.9) | 0.565 |  |

cpm – counts per minute ^a^Statistical test performed: mann-whitney U test
^b^Statistical test performed: t-test for independent samples
